# Barriers to help-seeking from healthcare professionals amongst women who experience domestic violence - a qualitative study in Sri Lanka

**DOI:** 10.1186/s12889-022-13116-w

**Published:** 2022-04-11

**Authors:** Tharuka Silva, Thilini Agampodi, Maggie Evans, Duleeka Knipe, Abey Rathnayake, Thilini Rajapakse

**Affiliations:** 1 South Asian Clinical Toxicology Research Collaboration, Faculty of Medicine, University of Peradeniya, Peradeniya, Sri Lanka; 2 Department of Psychiatry, Faculty of Medicine, University of Peradeniya, Peradeniya, Sri Lanka; 3 Department of Community Medicine, Faculty of Medicine and Allied Sciences, Rajarata University, Anuradhapura, Sri Lanka; 4 Centre for Academic Primary Care, Population Health Science Institute, University of Bristol, Bristol, UK; 5 Bristol Medical School, Population Health Sciences, University of Bristol, Bristol, UK; 6 Department of Sociology, Faculty of Arts, University of Peradeniya, Peradeniya, Sri Lanka

**Keywords:** Domestic violence, Healthcare professionals, Health services, Barriers to help seeking, Qualitative study, Sri Lankan women

## Abstract

**Background:**

Domestic violence (DV) is a major global public health problem which is associated with significant adverse consequences. Although Sri Lankan women who experience DV receive treatment from healthcare professionals (HCPs) for DV related physical and psychological problems, disclosure of DV within health services is quite low. This study explored barriers to disclosure of DV to HCPs among Sri Lankan women who experience DV.

**Method:**

This qualitative study took place in the Central Province of Sri Lanka. Twenty women who had experienced DV were recruited from Gender Based Violence Centers (Mithuru Piyasa Centers) and a toxicology unit of the two selected hospitals. Participants were purposefully selected using maximum variation sampling technique. In-depth interviews were conducted until data saturation was reached. Interviews were recorded, and analyzed using thematic analysis.

**Results:**

Survivor related barriers to help seeking included women’s lack of knowledge and perceptions about the role of HCPs***,*** lack of confidence in HCPs, fear of repercussions, personal attitudes towards DV, and their love and loyalty towards the perpetrator. Women preferred it if HCPs initiated discussions about DV, and they valued it when HCPs could be confidential and protect their privacy, and give enough time for DV related issues during consultations. A perpetrator related barrier was the controlling behavior of the perpetrator. Social stigma and social and cultural norms about the role of women emerged as the socio-cultural constraints to disclosure.

**Conclusions:**

Barriers to help seeking for DV from HCPs exist at individual, healthcare level, and societal level. Community programs are needed to increase women’s access to healthcare services and interventions should be implemented to develop effective, preventive, and supportive strategies at the healthcare system level.

**Supplementary Information:**

The online version contains supplementary material available at 10.1186/s12889-022-13116-w.

## Background

Domestic violence (DV), defined as “any act of gender-based violence that results in, or is likely to result in, physical, sexual, or psychological harm or suffering to women, including threats of such acts, coercion, or arbitrary deprivation of liberty, whether occurring in public or in private life” is a serious human rights abuse [1]. DV against women has become a global public health problem which comprises long term negative physical and mental health consequences for survivors. It also affects the healthy development and well-being of children and families and prevents women from participating fully in different areas of their lives such as family, community and society [2–4].

According to the latest World Health Organization (WHO) report, globally 26% of women aged 15 years and older have experienced physical and/or sexual violence from a current or former husband or male intimate partner at least once in their lifetime. In South Asia, the prevalence for women aged 15 – 49 years is 35%, which is higher than the 20% reported in high income countries [5]. In Sri Lanka, a South Asian country, there is a reported high prevalence of DV ranging from 17 – 72% [6–14]. The most recent national survey in Sri Lanka also reported a high lifetime prevalence of 40% in ever-partnered women, despite high levels of female literacy and gender parity [15, 16].

Previous studies from Sri Lanka have identified the negative consequences of DV on women’s health and wellbeing. These include physical consequences such as head injuries, black eyes, contusions, abrasions, lacerations, and burns, as well as psychological and mental health consequences such as lowered self-esteem, suicidal ideation, and suicide attempts as a result of DV [17–21]. In addition, evidence shows that pregnant women exposed to DV are more vulnerable to complications during labour and childbirth, and experience more adverse outcomes for their newborns and themselves [22].

Asian women are inclined to stay silent about their abuse experiences due to certain cultural values, fear, lack of knowledge and negative attitudes about appropriate sources of support services, lack of professional support, acceptance of abuse, and stigma [23–26]. Reasons given by Sri Lankan women for not seeking help for DV include, not knowing their options, being embarrassed, ashamed, fearing they would not be believed or would be blamed, concern about family reputation, thinking the violence was normal or not serious enough to seek help, and lack of knowledge of available formal support services [7, 9, 21].

As a formal source of help for DV victims, healthcare professionals (HCPs) such as doctors, nurses, and midwives, could play a major role in combating DV, as they are often the first point of contact for DV related physical and mental health problems [3, 26, 27]. However worldwide the majority of abused women, including Sri Lankan women, have used informal services such as parents, in laws, siblings, friends, and neighbors, rather than formal services such as police, health care providers, counselors, and other social services [7, 21, 28, 29].

Evidence shows that Sri Lankan women who are experiencing DV are inclined to approach hospitals for other complaints, such as sleep disturbance, loss of appetite, headaches, and self-harm attempts, rather than the violence itself [7, 20]. Among women who experienced violence by a partner in Sri Lanka, only a small proportion have sought help for the violence from hospitals and healthcare centres [7, 11, 21, 30]. However, even though almost all (97.7%) women who have been hurt to a degree that requires health care have received treatment for their injuries, less than half of them disclosed to the HCP that the injury was caused by their partner [21].

It is important to understand the barriers women face in seeking help from HCPs, in order to increase their access to healthcare settings to seek help for DV, to improve women’s personal attitudes and beliefs regarding help seeking for DV, as well as improving the approachability and responsiveness of HCPs’ to DV. Previous research has not explored the barriers to help seeking from HCPs among women who experience DV in Sri Lanka. Further, qualitative studies are needed to obtain a deeper understanding about this issue from the women’s point of view. Thus, the current study exclusively examined these barriers. The main aim of this study is to understand both internal and external barriers which prevent women’s help seeking from HCPs for DV; and in doing so, help to inform interventions at the individual and health care level, to minimize the harm and burden of DV in Sri Lanka.

## Method

### Study design and study setting

This qualitative study took place in the Central Province of Sri Lanka which has a population of 2.6 million, and is made up of three main districts; Kandy, Matale, and Nuwara Eliya. The population is a mixture of Sinhalese (66%), Tamil (23.8%), Sri Lankan Moors (9.9%), and other ethnics groups (0.3%). Men and women comprise 47.8% and 52.2% of the total population. In addition, 70.6%, 18.9%, and 10.5% of the population in the Central Province are classified as rural, estate, and urban respectively [15].

Previous studies have shown that the Central Province has a high prevalence of DV [9, 20, 22]. Service for DV is provided through walk-in gender-based violence centers (Mithuru Piyasa centres) situated at hospitals which provide in-hospital and/ or out-of-hospital services to women experiencing DV [31]. There are six Mithuru Piyasa centers in the Central Province.

### Participant recruitment

Women were recruited from two settings. The first consisted of Mithuru Piyasa centres at the teaching hospital in Kandy and the base hospital in Gampola. The second setting was the toxicology unit (ward 17) at the teaching hospital in Peradeniya, where women are admitted for medical management of deliberate self-poisoning, and where a high prevalence of DV has been reported [20].

Inclusion criteria were women aged 18 years or over experiencing current or past history of DV, including all forms of abuse, and DV perpetrated by a partner or a household member, who presented to a study setting between August 2019 and January 2020. Participants who reported a current psychiatric disorder diagnosis, based on self-report, were excluded from the study. Women presenting to the Mithuru Piyasa Centres during the study period, who met the inclusion criteria were considered eligible for inclusion in the study. Women presenting to the toxicology unit were initially screened for exposure to DV using the ‘Humiliation, Afraid, Rape, and Kick (HARK) 4-item questionnaire’ [32]. This questionnaire has been previously used in Sri Lankan research [20]. Women who screened positive for DV were considered eligible for inclusion in the study.

The investigator involved in interviews approached the participants in a friendly empathetic manner. She explained the purpose of conducting the research and details about participation. Participants were given an opportunity to ask questions and discuss any concerns prior to agreeing to take part. They were informed that we are asking them to share with us some personal and confidential information and therefore, if they feel uncomfortable talking about some of the topics, they do not have to answer the questions, and they do not have to give us any reason for not responding to the questions during the interview. Women were informed that refusing to participate in this study will not affect their treatment at the hospital in any way. Anonymity was also explained, whereby the names of participants would not be included in the analysis or reporting. In the experience of the authors, most women approached for participation in research studies welcomed the opportunity to talk about their DV experiences in a confidential, empathic, and non-judgmental setting where they are assured of no repercussions from professionals or family members. Womens’ participation in this research was entirely voluntary. Following good ethical research practice, written informed consent was obtained from all participants. Women were also informed of their right for self-determination, whereby they may withdraw from the study during or after it has taken place, without losing any of their rights as a patient in the hospital. It was made clear that the research team was independent of the HCPs and that no information would be shared with the HCPs or any other person outside the research team.

Since this study was a qualitative study, participants were purposefully selected to ensure a range of ages and localities, using maximum variation sampling technique. In maximum variation sampling*,* study participants are purposefully selected with a wide range of variation on dimensions of interest and it ensures participants with diverse background, views and perspectives are represented in the sample [33].

### Data collection

All in-depth interviews were conducted by the lead author using a topic guide created for the study, which was modified as needed after the first few interviews (see Additional file 1). Socio-demographic data were also collected using a brief questionnaire. Participants were asked about their help-seeking behaviour and barriers which prevented them from seeking help from health care services. A majority of the participants in this study were those who had already presented to healthcare services to seek help for DV but not until they had experienced abuse over a long period of time. Thus, to identify barriers which prevent them from seeking help, they were asked why they had not sought help at an earlier consultation. The researcher encouraged the women to talk freely and describe their experiences using open-ended questions, as well as probing questions to elicit more information pertinent to the research aim.

Women were interviewed in the local language in a room at the hospital with only the interviewer present. Interviews were conducted at a convenient time for the participant and hospital staff, so as not to interrupt or delay the woman’s treatment. If a woman became distressed during the interview, she was offered support and if needed referred to local psychiatry services.

Each interview lasted between 20 and 60 min. Interviews were conducted until data saturation was reached [34]. All the interviews were audiotaped, transcribed by an external transcriber, and checked for accuracy by the lead author.

During the interviews, all participants were assigned a code which was used to pseudonymize individual interviews and transcripts. Consent forms signed by individual participants did not have the code written on them. The link between participants’ names and codes were only known by the main researcher. The information recorded was kept confidential, and no one else except the researchers of this study have access to the information documented during the interviews. The information collected as paper copies were stored under lock and key, while the electronic data can only be accessed with a secure password. This password was known only to the researcher.

### Data analysis

Data analysis was undertaken in parallel to data collection using thematic analysis [33]. Twenty transcripts were independently coded by two investigators for verification, and a preliminary codebook was designed on the consensus of the two investigators. The code book was applied to all the transcripts using an iterative process of revision and re-coding as necessary. Themes and sub-themes were identified using an inductive approach, grounded in the data [36, 37]. Data analysis was conducted using the local language transcripts to minimize data loss in translation. Selected quotations which illustrate the emergent themes were translated into English by the lead author (who is a native bilingual speaker) and checked for accuracy by another native bilingual speaker.

### Quality assurance

Independent coding of all the transcripts by two investigators were done to increase credibility. To ensure the dependability and confirmability of the findings the analysis process was reviewed by the wider study team, including qualitative experts. After the two investigators finished the independent coding and making the code book, the coded transcripts and the codebook were reviewed for accuracy by qualitative experts. In addition, maximum variation sampling was used to enhance transferability.

## Results

The sample consisted of 20 women between the ages of 21 and 52 years (mean = 37 years). A majority of the participants were Sinhalese. Twenty-two women who presented to the two Mithuru Piyasa centres were approached and four declined to participate. Five women were approached in the toxicology unit and two of them took part in the interviews after they screened positive for DV exposure.

A majority of the women were married and nearly half of them were living with their partner. Almost all the participants had children. Seven women were employed, and thirteen were unemployed. Eighteen of the women had secondary education while 2 only had primary education.

**Fig. 1:**
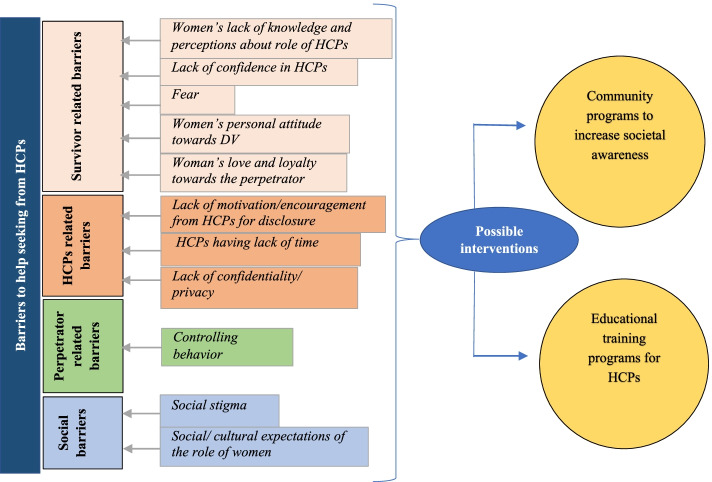
Barriers to help seeking from HCPs for DV and possible interventions

Themes emerged from the data that contributed to our understanding of both facilitators and barriers to help-seeking. The focus of this article is on barriers to help-seeking for DV encountered by women. Facilitators to help-seeking and the implications for service development will be published separately in a companion article. All women reported that they did not disclose the DV to HCPs after the initial abuse and discussed the reasons why they did not seek help/ disclose to HCPs earlier. Therefore, these barriers were reported retrospectively. Barriers to help seeking/disclosing to HCPs were identified at the survivor level, HCPs level, perpetrator level, and societal/ cultural level (Fig. 1).

### Barriers at the survivor level

#### Women’s lack of knowledge and perceptions about role of HCPs

One of the main barriers to accessing healthcare service was that women experiencing DV did not know or were uncertain that this was part of the role and responsibility of HCPs, and they were unaware of the available support services in the healthcare setting. One woman said:

“I haven’t heard that such problems [DV problems] can be disclosed to HCPs…I mean, I never thought of disclosing to HCPs and never thought that [I] can get help [from HCPs].” (SW13)

One of the participants, who was a member of hospital support staff herself, described how her personal situation led to her view that the role of HCPs did not extend to helping with DV:

“It’s like this for me…. Even though I have problems at home, I feel good when I come to work. On the other hand, my hometown is far away, I attend to work from elsewhere. I don’t think that problems at my home can be solved by disclosing to them [HCPs] due to the distance. The HCPs won’t be able to help me from here...So even if I disclose my problems, they can do nothing.” (SW10)

In addition to the hospital setting, participants were also asked how they felt about disclosing DV to the public health midwives (PHMs). PHMs are primary care field HCPs who provide the maternal and child health services and sexual and reproductive health services (SRH) at the community level. They are often the first contact HCP for women in the community, and often have a good rapport with them. Participants believed that in regards to PHMs too, DV was not part of their remit.

“I wish to talk with the midwife because we often meet…. but to disclose to her, I don’t know whether I can talk about these things with her. You know they look into problems such as pregnancy. Therefore, I haven’t disclosed to the midwife.” (SW13)

#### Lack of confidence in HCPs

Perceived lack of confidence in HCPs’ ability to help them was another reason for not disclosing. The level of confidence varied between types of HCP. For example, one woman mentioned that PHMs should give more attention to identifying and helping DV survivors compared to other HCPs in the hospital. She explained:

“It is because usually the PHM is the one who is more familiar to us. She knows about our family background. But we meet a doctor only on the day when we are getting treated or admitted to the ward. But we know the midwife for a long time.” (SW05)

Women said they were fearful of talking to doctors about DV owing to a perceived power hierarchy. Women preferred to talk to the PHM or a nurse.

“I would like to talk about my problems not with a doctor but with a nurse…Sometimes I feel scared of the way doctors ask some questions…I think the doctors ask some questions in a strange way... I don’t know how to explain it exactly…” (SW03)

Previous negative experiences in seeking help from HCPs prevented disclosure. When they did not get as much support as they had hoped for they lost confidence in asking for help again. One woman said:

“Actually, I had lot of problems those days, I tried to seek help from HCPs. Even the doctor could not give me a solution. So, I did not seek help anymore.” (SW12)

#### Fear

Some women were afraid of the perpetrator’s reaction to her disclosure, and they were afraid that the abuse would escalate if the perpetrator found out*.* Some women were also afraid of other people becoming aware of the disclosure to HCPs.

“His [husbands] friends will tell him that ‘she has gone to meet a HCP, we saw her at that place’ and then when he [my husband] gets to know that I have told someone else about this, there will be a fight. He will say, ‘ah you have told that person’, and the fight will be worsened.” (SW09)

Because they were worried about the outcomes that could emerge from disclosure, women refrained from going to hospital even after being physically injured.

“He hit me… I was severely bleeding. My mother asked me to go to the hospital. I was afraid because it will be a big story there again. I said it’s ok and I can bear it.” (SW05)

#### Women’s personal attitude towards DV

Some women remained silent because of their limited understanding of DV and their attitude towards it. Attitudes towards DV varied between the women. Some were silent because they considered the abuse to be not severe. One woman said, describing how she did not seek help till late.:

“One day he [my husband] hit me and thereafter I got a severe headache and vomiting...therefore I went to the hospital…That day too I might wait without going to the hospital [for help] if I didn’t develop a severe headache. That day I went to the hospital and disclosed because I couldn’t bear it up anymore...” (SW13)

Another woman reported that she did not consider the abuse as a sufficiently serious issue to seek help from HCPs. She believed that she could only seek help for DV if it became so bad that she was unable to go to work. She stated:

“Even though I had problems when I am at home, those problems didn’t cause difficulty for me to go to work or to do my work…I didn’t think about these problems while doing my work and I did my job well. I felt good [at work]. Therefore, I didn’t think of disclosing to a HCP.” (SW10)

#### Woman’s love and loyalty towards the perpetrator

A few women expressed their love and loyalty towards their husband by taking the decision not to disclose his abusive behavior, in order to prevent him from getting into trouble. One woman reported:

“then I thought that, no matter what he [husband] was doing to me, he might also get into trouble if I talk about this to a HCP. So that is why I was afraid to tell the hospital at the village…” (SW13)

### Barriers at the HCPs level

Barriers to accessing healthcare services to seek help for DV existed not only at survivor level, but also at HCPs level. Three categories could be identified, as described below.

#### Lack of motivation/encouragement from HCPs for disclosure

The major barrier described by women at the HCPs level, regarding their decision to seek help for DV, was wanting the HCPs to initiate the discussion. Study participants considered this to be very important in influencing them to disclose, instead of HCPs waiting for the women to initiate the conversation. One woman said:

“No one asked me about my personal problems…. if someone asked me, I could tell my problems...I could tell this and make my mind free...” (SW01)

One woman indicated that questions regarding DV are not asked routinely at SRH clinics. Her view was that HCPs in the SRH setting only focus on the duties of the clinic which do not include asking women about how things are going at home. This indicates that women tend to stay silent about their abuse at the SRH setting until HCPs initiate a discussion about possible abuse with them, and suggests that identifying and responding to women who are experiencing abuse could also be a part of their role. She explained:

“Such questions are rarely asked in antenatal clinics...they [HCPs in clinics] are mostly concerned about the children, they rarely ask about husbands. I think they are not really aware of it and do not carefully ask like our own doctor [HCPs in hospital]...” (SW06)

As reported by another woman, HCPs did not look at her problem from the DV perspective and did not ask her anything beyond giving medical treatment for the perpetrator’s drug addiction. The opportunity to disclose her abuse experiences to the HCP did not arise.

“Once I took him [husband] to a doctor and he got treatment for his drug addiction. But I didn’t tell the doctor about these problems…The doctor gave medicines for his drug addiction and did not ask me anything beyond that. Therefore, I also didn’t think of disclosing my problems to the doctor…” (SW15)

#### Time constraints in the healthcare settings

HCPs not having enough time to discuss such issues with women was another barrier. Women said that there was lack of time to talk with HCPs particularly in the SRH setting. One woman said:

“At the antenatal clinics we don’t get enough time to talk with them [HCPs].... therefore, I haven’t talked to them about this problem.” (SW07)

One woman preferred to talk with a HCP in the hospital, as they had more time than in the SRH clinics. She explained:

“It is hard for them [HCPs in SRH setting] because they don’t have enough time there to talk with women. If a woman wants to talk to them, she can meet them [HCPs in SRH setting] separately at another time, it is ok. If not, it is better to go to the hospital...” (SW06)

#### Lack of confidentiality/ privacy

Women said there was not enough privacy to talk about DV with HCPs. They preferred to stay silent as they were too embarrassed to talk in front of other patients, HCPs or family members. The Outpatient Departments (OPD) in Sri Lankan hospitals are usually quite busy and crowded with inadequate space. Therefore, during medical consultations, most often there will be not only the HCP and the patient, but also other HCPs and patients in the same room.

“We feel ashamed and worried when they [HCPs] ask in front of other people. I would prefer if they talked privately when we tell something personal. Even though someone has a problem they don’t like to tell (like that) and will try to hide when the HCPs ask in front of everyone. I’ve experienced it several times and I’ve seen some other women also face the same situation… I feel ashamed to talk about it and I am trying to hide that I have this kind of problem in front of everyone. But I would certainly like to reveal my problems privately…” (SW17)

“There were a lot of people around when the outpatient department (OPD) doctor asked everything about my problem. I felt it would be good if only the doctor was there because there were lot of people in the OPD. I felt helpless in front of a lot of those people…” (SW13)

Women discussed how they were trying to hide their actual situation and trying to save their self-respect because of the lack of privacy in the health care setting.

“I don’t like if someone is there when I disclose to the doctor... we do also have a self-respect and feel shame... Letting someone else know about your personal matters is a big deal, it is a difficult thing. So, if it is discussed only between the doctor and the patient, it is ok.” (SW06)

### Perpetrator related barriers

#### Controlling behavior

In addition to survivor related barriers and HCPs related barriers, another important constraint for women’s disclosure was the perpetrator’s controlling behavior. One woman explained how her husband tried to prevent her from going to the hospital after she was severely injured by his abusive behaviour.

“He [husband] told me not to go to the hospital and to take medicine from the nearby dispensary...he told me that the injury is not very serious and to tell them [HCPs] that it is just an earache and take medicine...” (SW05)

Another woman stated that her husband and his family did not allow her to talk or meet anyone outside the home and therefore she didn’t get any chance to seek help for DV. She said:

“My husband and his family don’t want me to talk with anyone. Therefore, I secretly went to meet even the midwife in our village... I was afraid to go to her because she was a friend of my husband… they were relatives…I was afraid that she will tell my husband if I tell her about these problems…therefore I didn’t tell anyone about anything when I was in Colombo with my husband…” (SW17)

### Socio-cultural barriers

Societal/ cultural barriers including social stigma and expectations of the role of women were another major barrier for women to seek help from HCPs.

#### Social stigma

Women also did not want to be stigmatized, or face disapproval or disgrace from others including HCPs for talking about DV. They thought that DV was too personal matter to talk about with others. Thus, they tended to be silent about the abuse and sometimes tried to solve the problem within the house without disclosing to others, including HCPs.

Quoting this, one woman said she did not disclose to HCPs about her abuse experience because she felt too shy to talk about it and did not want to be labeled or judged. She said:

“I feel ashamed to disclose to them [HCP]. I am worried about what the doctors may think of me if I tell them these things …. sometimes they may think that I am behaving like I am the only one who is having these problems… therefore most of the time I am trying to hide my problems…” (SW17)

Another woman reported that there are some personal things women should not disclose to HCPs.Women should be careful when disclosing personal information.

“There are times that we can’t tell about our personal problems to HCPs. Therefore, we must use our brain and think whether we should tell this or not. Sometimes there are problems - like people fight because of men having extra marital affairs. People have such problems at home. We should first think whether we are going to tell this to the HCP or not” (SW06)

“I’ve gone to the clinics with my children to get them vaccinated. Our midwife is a nice person… I don’t see anything bad about her… But I didn’t tell my family problems with her because I don’t like to tell my family matters outside.” (SW16)

#### Social / cultural expectations of the role of women

Some women described how they did not seek help from HCPs because they put their family before themselves. They chose to be silent because of their need, first and foremost, to be a good mother and fulfill their responsibilities to their children. This shows that social/ cultural expectations about the role of women have prevented women from disclosing DV, and it has caused women to endure abuse and stay in an abusive relationship. One woman explained:

“Hmm… I was not much concerned about my husband’s behavior and the things I had experienced. I was only thinking of my four children. I did my job to educate them, and I looked after my children well. I did everything that was needed for my children. I was suffering alone from inside...” (SW10)

Also, they wanted to have a good family life and therefore they tried as much as possible to keep their family together. One woman said:

“I didn’t think of disclosing... My ex-husband also left me for something I never did. He [ex-husband] also regrets it now, recently my elder son went to meet him, he [ex-husband] cried a lot. So, he regrets that he left a good woman like me. With the second husband, instead of going from place to place to ask for help, I tried to make my family life better, without telling anyone...” (SW12)

While the women were focused on fulfilling their traditional gender role as a wife and a mother, they did not have enough time to think about themselves. Women have little freedom to take care of themselves since earning money and taking care of children and husband, comes first. Thus, having lack of time to access healthcare services also created a barrier for women to seek help from HCPs.

“I didn’t have time to disclose it. I had to go to work while my child was in daycare. We lived in a rented house. Always we lived in a rented house. I left the child in the daycare, I went to work and came back in the evening. When I came home in the evening, he [husband] was drunk or he hadn’t come home, so some days the child was not picked up from the daycare. Such things happened. With all that work, I didn't have time to go...” (SW09)

## Discussion

This study elaborates the real life scenarios as to why women experiencing DV in Sri Lanka endure the abuse without seeking help from HCPs. We identified multiple barriers operating at different levels: individual, healthcare, perpetrator, and societal level. We observed that women might disclose indirectly to HCPs or at a point of increase in the severity of the abuse.

The present study supports previous findings that women are not aware that HCPs could be involved in DV related issues and provide support, [9, 21], apart from some women who seek help from healthcare services mainly for their security and safety [8]. Women also reported lacking confidence in HCPs’ ability to help with DV, particularly doctors, arising from previous negative encounters, and the fear that HCPs may disclose to family members and police [40–42]. Further, the present study identifies many personal barriers that prevent women from disclosing to HCPs, including fear of repercussions, not believing their abuse to be serious, and their love and loyalty towards the perpetrator. These findings are consistent with most of the studies worldwide [25, 38, 39]. In addition, there is a discrepancy between different womens’ personal attitudes, perceptions, and knowledge about the role of the HCPs. While some women prefer and feel confident to talk about DV experiences with certain HCPs, some women appear not to be aware that responding to DV is a part of the role of the HCPs. This reflects a varying understanding among women about the role of HCPs, and PHMs in particular, with regards to the provision of support for DV. Such misconceptions may prevent women from seeking help in situations when they face DV, even if they do have contact with a primary healthcare worker such as a PHM. This is a significant lost opportunity for provision of support, especially since Sri Lanka has a widespread primary healthcare system that reaches to grassroot levels, often via PHMs and the primary healthcare team. Therefore, provision of information and education for the community and all women in general is important.

Other barriers relate specifically to HCPs. These barriers include lack of motivation or encouragement from HCPs to disclose, such as HCPs’ failure to initiate the discussion, lack of time in HCP consultations, and lack of confidentiality and privacy. Other studies have also shown that women wished HCPs to ask them about DV [26, 40, 43]. Women in this study discussed the importance of HCPs being able to understand and recognize indicators of DV, but they also recognized the heavy workload and lack of time that prevents HCPs giving priority to DV related issues in medical consultations [27, 44].

Many Sri Lankan women try to fulfill perceived social expectations of their role as a wife and a mother, allowing them little freedom to take care of themselves, since earning money and taking care of children and husband take priority. One study shows that being previously stigmatized and judged by HCPs when attempting to disclose DV, prevents women from trying again [7]. Owing to fears of social stigma, abused women are reluctant to disclose personal issues to outsiders and they prefer to seek help mainly from informal sources such as friends and relations, rather than from formal sources [30]. This finding is consistent with most of the international studies [28, 45].

Women’s help-seeking from HCPs was often inhibited by their husband (usually the main perpetrator). Even when a woman was in a serious condition due to the abuse, the perpetrator used controlling behavior to prevent her from going to the HCP to seek help. In line with patriarchal cultural norms, Sri Lankan men often believe that women should obey their husband, and cultural norms support the attitude that men are the decision-makers in the family [22]. The wider family may also inhibit disclosure of DV, following cultural norms of the importance of maintaining the marriage. Disclosure may result in the husband being sent to jail if the hospital informs the police [18]. In this study women also expressed love and loyalty towards their husbands and did not want to cause them trouble by disclosing their abusive behavior.

### Research, practice, and policy implications

The findings of the current study clearly suggest the need for a three-way approach to address DV (Fig. 1). Empowerment of women would be one of the most important strategies as this study shows that women keep silent due to many socio-cultural reasons. Enhancing skills of HCPs would be highly beneficial, as the study findings reveal the importance of building trust and improvement of soft skills. Increased awareness and concern on the part of the HCPs to identify DV, would also help women talk about the issue. Thirdly, health and social policies could be newly implemented or strengthened, by adopting an inter-sectoral action involving health, education, social services, and religious sectors.

Women should be informed and educated about DV, its consequences, and the available support services in the healthcare setting. Successful approaches include using “positive role model stories” based on true stories that emphasize safe outcomes from disclosing to HCPs [46] as well as mass media and community awareness campaigns focusing on family relationships [47]. SASA! is another community mobilization approach designed by Raising Voices, for African communities and currently being used in many countries around the world to prevent violence against women, which is aimed to change social norms and attitudes that lead to power imbalance between women and men, using a four-phased structured program of discovery, critical reflection, and skill building among the communities [48]. SASA! was significantly associated with lower DV prevalence as well as changes in attitudes and norms within the communities where it has been implemented [48–50].

HCPs should also receive training on how to identify DV survivors and respond confidentially, as well as improving understanding of what kinds of support are needed. Low-cost interventions that have been successful for other health-related conditions may be transferable to DV. For example, a successful intervention was conducted to promote exclusive breast feeding among Sri Lankan mothers who attended antenatal clinics, by training and educating PHMs to provide counselling for mothers with breastfeeding problems and conducting health education programs for pregnant mothers [51]. In addition, HCPs should be encouraged to routinely screen women and provide appropriate support for DV in healthcare settings [52]. Developing methods to screen women for DV, such as developing a screening tool is recommended to address the issue [53, 54].

Lack of time and heavy workload are major barriers in service provision and effort should be made to improve the quality of healthcare services. Confidentiality and privacy issues are major ethical problems that need to be addressed immediately by national and local health authorities as well as medical education experts.

## Strengths and limitations

### Strengths

Although a few local studies have been conducted previously to explore help seeking behaviors of women experiencing DV [7, 9, 11, 21, 55], none of these studies have examined the barriers to HCP-related barriers to help seeking from HCPs in a detailed manner. The current study explores this complex area further by thematic classification of barriers enabling us to draw out different broader intervention approaches that would be helpful in supporting women to seek help from HCPs. The qualitative nature of the study will give in-depth insight that will be helpful in the detailed planning of intervention programmes. We utilized robust analysis techniques, such as independent coding of interview transcripts, designing of the codebook, and identification of themes by two investigators for verification in order to give a deeper understanding of the participants’ experiences.

### Limitations

The study participants had all approached HCPs to seek help for DV. The sample did not include any non-help-seekers, although women described how they did not seek help in the earlier instances and therefore would partially resemble this group. The credibility and trustworthiness of data would have been improved if the study adopted triangulation by interviewing other key informants such as HCPs or family members.

## Conclusions

Barriers to help-seeking for DV from HCPs exist both internally within the survivors, and externally in HCPs and the socio- cultural context. The gap between women who experience DV and HCPs should be bridged, in order to minimize the burden of DV in Sri Lanka, by implementing community programs to inform and educate both women and perpetrators regarding DV, its negative consequences and services that are available in the healthcare system. Training programs are needed to improve HCPs’ response to DV. In addition, tried and tested context specific social interventions are needed to improve community support to minimize DV in Sri Lanka.

## Supplementary Information

**Additional file 1.** Topic guide to interview women who experience DV.

## Data Availability

Because of the difficulty of anonymizing qualitative data and the risks this poses to survivors, this study does not make data available. The data that support the findings of this study are available from the corresponding author upon reasonable request.

## Abbreviations

DV: Domestic Violence
HCPs: Healthcare Professionals
IPV: Intimate Partner Violence
HARK: Humiliation, Afraid, Rape, and Kick
PHMs: Public Health Midwives
SRH: Sexual and Reproductive Health Services
OPD: Outpatient Department
