# Supplementary material for: Barriers to help-seeking from healthcare professionals amongst women who experience domestic violence - a qualitative study in Sri Lanka
Source: BMC Public Health. 2022 Apr 11;22:721. doi: 10.1186/s12889-022-13116-w (PMC9004164; doi:10.1186/s12889-022-13116-w)
Supplement: Supplementary file 1 — Additional file 1. Topic guide to interview women who experience DV. [file 12889_2022_13116_MOESM1_ESM.pdf]

Additional file 1: Topic guide to interview women who experience DV.

Participant ID No :
Time started :
Time ended :

1. Date of interview (day/month/year):	_______/_______/________
2. Date of 1st contact with the service	_______/_______/________
3. GBV centre/ Inpatient ward 17 /community/Other (describe):	
4. How old are you?	_____
5. What is your ethnic background?	0 Sinhala<br>1 Tamil<br>2 Moor<br>4 Burger
6. What religion are you?	0 Buddhist<br>1 Hindu<br>2 Christian<br>3 Muslim<br>4 No Religion
7. Where do you live? (area/ town)	___________________
8. What language that this interview is conducted	0. Sinhalese<br>1. English<br>2. Tamil
9. Are you married or not?	0. Single<br>1. Married<br>2. Separated/ divorced<br>3. Spouse dead<br>4. In a relationship/ has a boyfriend or partner
10. IF married;<br>How long have you been married for?	
11. IF married;<br>Was it an arranged marriage or your choice?	0. Arranged<br>1. Your choice
12. Do you live with your husband/ partner/ boyfriend?	0. No<br>1. Yes

13. Who lives within your house?	---<br>---<br>---<br>---<br>---<br>-----------------------------------
14.<br>i. Do you have any children?<br>If Yes;<br>ii. How many children do you have?<br>iii. How old are your children?	0. No<br>1. Yes<br>_______________________________<br>_______________________________
15. Are you working outside the house at the moment?<br>If Yes;<br>What is your job?<br>Is it full-time or part-time?	0. No<br>1. Yes<br>____<br>____
16. What is your highest level of education?	0-No schooling<br>1-completed between grades 1-5<br>2-completed between grades 6-10<br>3-Passed O/L<br>4-Passed A/L<br>5-completed university /postgraduate qualifications
17. Do you suffer from any psychiatric disorder? (depression, anxiety, etc)	0. No<br>1. Yes
18. Do you suffer from any chronic illness/disability/impairment that causes difficulty with your day-to-day activities? (difficulty in hearing/ seeing/ walking/ remembering/ self-care activities/ communicating)	0. No<br>1. Yes

HARK Screener (for the toxicology unit)

Within the last year…	Yes	No	Prefers not to say
19. Have you been afraid of your partner/ husband/ boyfriend or another family member who lives in your household?	1	0	
20. Have you been kicked, hit, slapped or otherwise physically hurt by your partner/ husband/ boyfriend or another family member who lives in your household?	1	0	
21. Have you been raped or forced to have any kind of forced (i.e. against your will) sexual activity by your partner/ husband/ boyfriend or another family member who lives in your household?	1	0	
22. Have you been humiliated or emotionally abused in other ways by your partner/ husband/ boyfriend or another family member who lives in your household?	1	0	
**SCREENS NEGATIVE →**	***DISCONTINUE INTERVIEW***		
**SCREENS POSITIVE →**	***CONTINUE INTERVIEW***		

We will be talking today about the abuse / violence / difficult relationship (choose suitable word) you are experiencing in your life, and your experiences of seeking help from health care professionals. I understand that it can be difficult to talk about so please take your time. If any of the questions make you feel uncomfortable just let me know. You do not need to answer if you do not want to, and be reassured that we can stop the interview at any time.

Q1: Can you tell me who the person is/ who is acting in this way? (Probe: husband, partner, in-laws etc).

Q2: How long has this been going on for?

Q3: Have you ever tried to get help or talked to anyone about the abuse/ violence?

If Yes:
Can you tell me about any occasion when you told somebody or asked for help about what is happening with (name the perpetrator eg: husband)

Probes: whom did you approach for help/ why did you go to them for help? What was their response?

If she hasn't asked for any help – why, what makes it difficult?

Q4: Have you ever talked to a health care provider about what is happening with (name the perpetrator) **IF'NO', NEVER DISCUSSED IT WITH A HEALTH CARE PROVIDER GO TO Q5)**

IF YES

a) Who did you talk to? (eg doctor, nurse, midwife etc)
b) Who told you to talk to a health care provider?
c) Where did you talk to him/ her?
d) Can you tell me about your experience of talking to a health care professional?

Probes:

What prompted you to talk to that person (e.g. health issues/something happened at home); What did you say to him/her; what do you think you were hoping for?; what did he/ she say / do after? Was there a referral or follow-up? How did you feel about his/her response, did you get what you were hoping for; how did you feel after talking about

the abuse / violence? Is there anything else you think the health care provider should have done?

d) Were you on your own at that appointment or was someone else there (eg: family member, perpetrator)

Probes:

Do you usually go to heath care appointments alone or does someone go with you? Does that make it difficult for you to talk? How do you usually travel there?

e) Have you ever tried to talk to a health care provider, but they didn't listen? - If yes ask for examples

Q5: IF NO, she never talked to a health care provider

Do you know who are there in the health setting to provide support/help for women who experiencing domestic violence?

If yes, who do you think can be help/support for women experiencing DV?; What did you feel (fear, shame, stigma); what were you afraid would happen; was there someone with you; what was the setting like? What was the health care provider like? Were you worried what people would think of you?

If No, why didn't you think to talk to a health care provider?; what type of health care provider should be responding to women experiencing DV? What did you feel (fear, shame, stigma); what were you afraid would happen; was there someone with you; what was the setting like? What was the health care provider like? Were you worried what people would think of you?

Q06: We've come to the end of the interview. Before we end I'd like to know.

a. Is there anything else you'd like to tell me?
b. How do you feel about our conversation today?
c. Would you find it helpful to talk to someone from [name of key referral organisation]? I can arrange this for you [Researcher – can organise via the relevant clinic provider]

Thank you for sharing your experiences and helping with our research. I know that it can be very difficult to talk about. It takes a lot of strength to be open with someone that you do not know, and I want you to know that I really appreciate it.
